# Tropomyosin isoforms encoded by *TPM2* control the actin-bundling activity of fascin-1

**DOI:** 10.1186/s40659-025-00640-3

**Published:** 2025-08-31

**Authors:** Małgorzata Siatkowska, Katarzyna Robaszkiewicz, Andrea Rousová, Jiří Navrátil, Lucia Knopfová, Gábor Talián, Petr Beneš, Joanna Moraczewska

**Affiliations:** 1 Department of Biochemistry and Cell Biology, Faculty of Biological Sciences, Kazimierz Wielki University, Ks. Józefa Poniatowskiego 12, 85-671 Bydgoszcz, Poland; 2 Department of Experimental Biology, Faculty of Science, Masaryk University, Brno, Czech Republic; 3 Department of Pathological Physiology, Faculty of Medicine, Masaryk University, Brno, Czech Republic; 4 International Clinical Research Center, St. Anne’s University Hospital, Brno, Czech Republic; 5 Department of Biophysics, Medical School, University of Pécs, Pécs, Hungary

**Keywords:** Fascin-1, *TPM2*, Tropomyosin, Isoforms, Actin, Bundling

## Abstract

**Background:**

In many types of tumors, the expression patterns of actin-binding proteins –fascin-1 and various isoforms of tropomyosin – are altered. Fascin-1 is an actin-bundling protein that promotes cancer cell motility, whereas tropomyosin functions as a tumor and metastasis suppressor. However, the mechanisms by which tropomyosin isoforms regulate fascin-1 remain poorly understood. This study aimed to investigate the reciprocal effects of fascin-1 and tropomyosin isoforms on their interactions with actin and on the formation of actin bundles.

**Methods:**

Recombinant fascin-1 and the cytoskeletal tropomyosin isoforms encoded by *TPM2* (Tpm2.1, Tpm2.3, and Tpm2.4) were expressed in BL21-DE3 cells and purified. High-speed centrifugation was employed to assess the actin affinities of fascin-1 and the Tpm2 isoforms. Actin filament bundling was analyzed using low-speed centrifugation and fluorescence microscopy. A pull-down assay was performed to examine direct interactions between fascin-1 and the Tpm2 isoforms. Confocal microscopy was used to analyze the localization of fascin-1 in the metastatic SAOS-2 LM5 cell line overexpressing Tpm2 isoforms.

**Results:**

Among the three recombinant, acetylated Tpm2 isoforms, Tpm2.4 exhibited the highest affinity for F-actin. All Tpm2 isoforms strongly inhibited fascin-1-mediated actin bundling at low fascin-1 concentrations, with bundling restored only at substantially higher fascin-1 levels. The resulting actin bundles contained both Tpm2 and fascin-1; however, the number of filaments per bundle was reduced in the presence of any Tpm2 isoform. Fascin-1’s affinity for actin was decreased in the presence of Tpm2 isoforms, and increased Tpm2 occupancy on actin filaments partially displaced fascin-1. In contrast, fascin-1 binding did not affect the affinity of Tpm2 isoforms for actin. Pull-down assays revealed that Tpm2 isoforms can directly interact with fascin-1, with Tpm2.4 showing the highest affinity. The inhibitory effect of Tpm2 on fascin-1–actin interactions was further supported by cellular data, which showed that overexpression of cytoplasmic Tpm2.1, Tpm2.3, or Tpm2.4 in SAOS-2 LM5 cells reduced fascin co-localization with actin.

**Conclusion:**

Cytoplasmic Tpm2 isoforms regulate actin bundling activity of fascin-1 by organizing protein composition in the bundles, a mechanism that may contribute to the suppression of metastatic phenotype in cancer cells.

## Introduction

Enhanced cell migration and invasion, key hallmarks of metastasis, depend on the dynamic polymerization and remodeling of actin architecture [1]. The initiation and elongation of actin filaments at the leading edge of migrating cells generate the force needed for the extension of cellular protrusions, such as lamellipodia, filopodia, and invadopodia. Meanwhile, the formation of stress fibers, contractile actin-myosin assemblies linked to focal adhesions, is crucial for contraction, retraction, and adhesion, all of which contribute to efficient cell migration [2, 3]. The remarkable functional diversity of actin filaments stems from their interactions with a wide array of actin-binding proteins (ABPs), which regulate their structural and functional adaptability in response to cell signaling [3–5]. Consequently, altered expression profiles of numerous ABPs are characteristic of various types of malignant tumors [2].

Fascin is a 55 kDa protein involved in the formation of actin bundles in filopodia, invadopodia, and the distal ends of stress fibers connected to focal adhesions [6–9]. The most widely expressed isoform, present in mesenchymal cells is fascin-1 [10]. It is a globular protein composed of four β-trefoil domains, with a deep cleft between domains 1–2 and 3–4, creating an internal pseudo twofold symmetry between the domain pairs. The fascin monomer contains at least two actin binding sites that can interact with parallel actin filaments, resulting in bundle formation [8, 11, 12]. In these bundles, actin filaments are cross-linked into hexagonally packed filaments with uniform polarity, providing mechanical stiffness to elongated cellular structures [13–16].

The access of ABPs to actin filaments is regulated by tropomyosin (Tpm), coiled-coil dimers that polymerize head-to-tail along the filament, forming continuous chains on both sides of actin. Tpm stabilize actin filaments and regulate their dynamics, organization, and accessibility within the actin filament network [17, 18]. Tpm constitute a family of approximately 40 proteins encoded by four distinct genes in humans (*TPM1*–*TPM4*). Structural diversity among Tpm isoforms arises from alternative promoter usage and exon splicing, which generate isoforms classified as high molecular weight (HMW) and low molecular weight (LMW) Tpm [17]. This structural variability underlies the functional diversity of Tpm isoforms, enabling differential regulation of proteins involved in actin nucleation, polymerization dynamics, filament bundling, and contraction. The mechanisms by which Tpm modulates actin's interactions with ABPs remain elusive. Although Tpm is traditionally regarded as a steric blocker that competes with ABPs for actin binding, several studies have demonstrated that certain Tpms can instead recruit ABPs or enhance their binding [19–21].

In many types of tumors, the expression levels of both fascin-1 and Tpm are significantly altered, correlating with disease onset and progression [22]. Depending on the tumor type and its aggressiveness, Tpm expression can be either upregulated or downregulated, suggesting that Tpm participates in distinct processes involved in oncogenic transformation and metastasis [23–26]. The HMW products of *TPM1* and *TPM2* are generally downregulated in transformed cells and have thus been identified as tumor and metastasis suppressors [24, 27–31]. In contrast, elevated fascin-1 expression is positively correlated with aggressive phenotypes and shorter survival [32–35]. During oncogenic transformation, fascin is implicated in the epithelial-mesenchymal transition, promoting a highly motile and invasive phenotype [33, 34, 36]. These findings emphasize the importance of fascin and Tpm expression in cancer progression and highlight their potential as biomarkers or therapeutic targets [30, 37–41].

The mechanisms underlying the Tpm-dependent regulation of fascin activity remain poorly understood. Skeletal muscle Tpm has been shown to strongly inhibit fascin’s interaction with actin filaments and its ability to cross-link filaments into bundles [6]. In contrast, neither HMW nor LMW Tpm isoforms from non-muscle rat cultured cells affected fascin’s binding to actin [42], unless their affinity for actin was enhanced by caldesmon [43]. Notably, in a cellular context, the non-muscle isoform Tpm1.7 was reported to recruit fascin to actin filaments, increasing its localization in filopodia [44].

To gain insight into the mechanisms governing Tpm-dependent regulation of fascin-1 (hereafter referred to as fascin) interactions with actin, we conducted in vitro analyses using recombinantly produced proteins. Specifically, we examined the regulatory properties of the HMW products of *TPM2*, which are differentially expressed in different cell types. The best-studied Tpm2 isoform is Tpm2.1. It is consistently associated with the actin cytoskeleton and plays a key role in regulating actin dynamics, although its localization to specific actin structures is context-dependent. It is widely expressed in fibroblasts, smooth muscle cells, skeletal muscle cells, and human epithelial cells, where it serves as a core component of stress fibers [45]. In osteosarcoma cells (U2OS), Tpm2.1, along with other Tpm isoforms, localizes to contractile stress fibers, including transverse arcs and ventral stress fibers, and is particularly enriched at the distal ends of dorsal stress fibers [46]. In contrast, in non-transformed breast epithelial cells, Tpm2.1 localizes to the basal membrane and is also present at the leading edge [47]. While Tpm2.3 and Tpm2.4 are less well characterized, they are classified as high-molecular-weight *TPM2* isoforms and are therefore likely to localize to stress fibers and other actin-rich regions. Interestingly, expression of Tpm2.3 has recently been confirmed in human hFOB osteoblasts by proteomic analysis, and siRNA-mediated downregulation of Tpm2.3 inhibits their differentiation, indicating its functional relevance in this process [48].

**Fig. 1:**
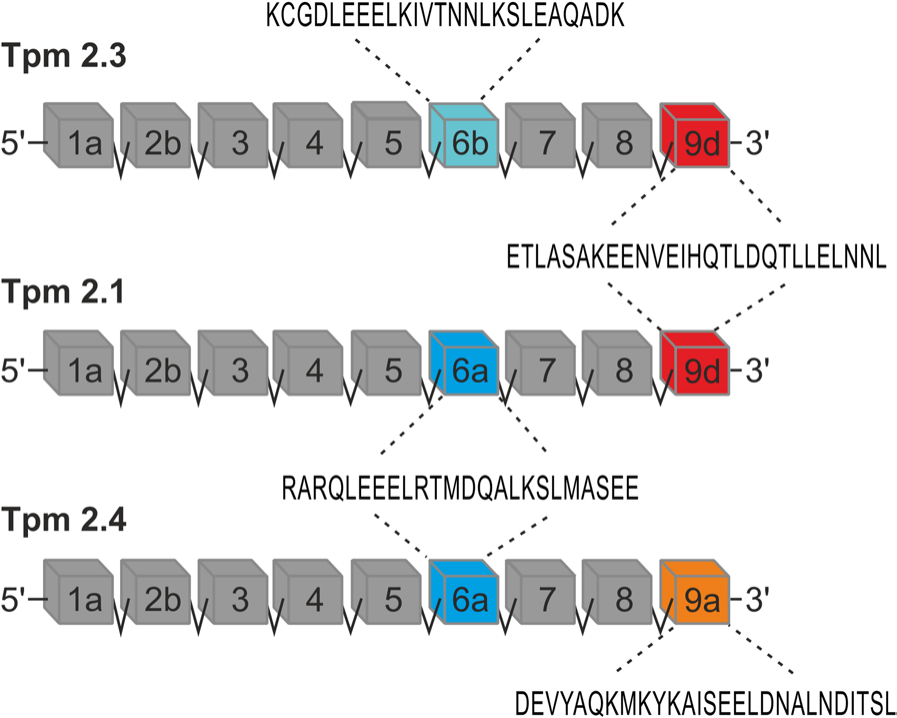
A diagram illustrating the exon composition of the *TPM2* gene, which encodes the non-muscle isoforms Tpm2.1, Tpm2.3, and Tpm2.4. Color coding indicates alternatively spliced regions, with dashed lines pointing to the corresponding amino acid sequences encoded by the alternative exons of human tropomyosins

In this study, the cytoplasmic Tpm2 isoforms – Tpm2.1, Tpm2.3, and Tpm2.4, generated through alternative splicing of exons 6 and 9 (Fig. 1), were compared to assess their distinct effects on fascin regulation and actin bundling. We found that cytoplasmic Tpm2 isoforms regulate fascin’s access to actin and its bundling activity through a complex mechanism involving both partial displacement of fascin from actin filaments and direct interactions between Tpm2 and fascin. At the cellular level, overexpression of Tpm2 isoforms reduced co-localization of fascin with actin in the highly aggressive, metastatic SAOS-2 LM5 osteosarcoma cell line. These regulatory mechanisms may contribute to the suppression of metastatic phenotypes in cancer cells.

## Materials and methods

### Expression and purification of recombinant acetylated Tpm2 isoforms

Plasmids pET with inserts encoding products of human *TPM2* gene: Tpm2.1 (NCBI Reference Sequence: NM_213674.1), Tpm2.3 (NCBI Reference Sequence: NM_001301226.2) and Tpm2.4 (NCBI Reference Sequence: NM_001301227.2) were designed using VectorBuilder. online tool: https://en.vectorbuilder.com/, with codons optimized for *E. coli*. DNA synthesis and cloning were outsourced to VectorBuilder Inc, Chicago, USA.

To produce N-terminally acetylated tropomyosins, Tpm2 isoforms were co-expressed with the fission yeast NatB complex as described in [49]. Briefly, BL21-DE3 competent cells (ThermoFisher, Waltham, USA) carrying pNatB (pACYCduet-naa20-naa25, Addgene, https://www.addgene.org/53613/) were transformed with plasmids encoding Tpm2 isoforms. Mid-log cultures in LB Broth were grown for 3–5 h with 0.4 mM IPTG. Cells were harvested by 20 min centrifugation at 4 °C, 11,000 rpm using a GSA rotor in a Sorvall RC-5B centrifuge. Pellets were resuspended in lysis buffer containing 50 mM Tris–HCl, 10 mM EDTA, 20% sucrose, 0.5 mg/mL lysosyme, pH 7.5, homogenized on ice, subjected to 1 M NaCl freeze–thaw cycle, and sonicated. Cell debris was removed by centrifugation and tropomyosins were purified by ammonium sulfate precipitation and FPLC using 50 ml of BioRad Macro Prep 25Q resin by elution with 0–1 M NaCl gradient. Fractions containing tropomyosin were dialyzed against 10 mM TRIS, 30 mM NaCl, 2 mM MgCl_2_, 1 mM DTT, 0.02% NaN_3_, pH 7.5.

Protein concentration was determined spectrophotometrically at 280 nm using molar extinction coefficient 11,920 M^−1^ × cm^−1^ for acetylated Tpm2.1 and Tpm2.3 and 17,880 M^−1^ × cm^−1^ for acetylated Tpm2.4. The coefficients were calculated from the tropomyosins amino acids sequences in the web.expasy.org/protparam/tool. The presence of acetyl group was confirmed by mass spectrometry (Environmental Laboratory of Mass Spectrometry, Department of Biophysics, Institute of Biochemistry and Biophysics, Warsaw, Poland).

### Expression and purification of recombinant fascin-1

DNA encoding mouse isoform of fascin-1 (NCBI Reference Sequence: NM_007984.2) was cloned into pGEX-6P-1 plasmid containing GST tag and PreScission protease recognizable site, between the BamHI and XhoI restriction sites. The plasmid was a kind gift from Dr. Feng Ching Tsai, CNRS, France. The GST fusion protein was expressed in Rosetta *E. coli* cells. Thirty milliliters of LB broth containing 0.05 mg/mL ampicillin were inoculated with a single colony obtained after transformation and incubated overnight at 37 °C with shaking at 220 rpm. The following morning, the overnight culture was added to 2.4 L of fresh LB broth with ampicillin, and incubation continued under the same shaking conditions until the culture reached an OD₆₀₀ of 0.5–0.8. The culture was then cooled to 26 °C. Protein expression was induced with 0.1 mM IPTG, and fascin was expressed overnight at 26 °C with shaking at 180 rpm. The following morning, bacterial cultures were centrifuged at 11,000 rpm using the GSA rotor in the Sorvall RC-5B centrifuge for 20 min at 4 °C. Pellets were resuspended in 25 mL of lysis buffer containing 50 mM Tris (pH 7.5), 300 mM NaCl, 1 mM EDTA, 3 mM DTT, 0.2 mM PMSF, 0.5 mg/mL lysozyme, 0.1 mg/mL DNase, 5 mM MgCl₂, 0.5% Tween-20. After homogenization and sonication, the lysate was centrifuged at 30,000 rpm in the Beckman Optima L-90 K ultracentrifuge, rotor 70Ti, for 20 min. The resulting supernatant was incubated with 5 mL of glutathione–agarose beads (Pierce, Thermo Fisher Scientific, Waltham, MA, USA) overnight at 4 °C with gentle mixing. The resin was washed seven times with 50 mM Tris–HCl, 150 mM NaCl, pH 7.5, using three-fold the resin volume for each wash. Fascin was cleaved from the beads by a 2 h incubation with 5 mL of cleavage buffer (50 mM Tris–HCl, pH 7.0, 150 mM NaCl, 1 mM EDTA, 1 mM DTT) containing PreScission Protease (GenScript, Piscataway, NJ, USA; 1.4 U/µL of protein). Following centrifugation, the supernatant was collected, and 4 mM Pefabloc was added to inactivate the protease. Samples were analyzed by SDS–PAGE. Pure fascin was dialyzed against G-buffer (5 mM Tris, pH 7.5, 0.2 mM ATP, 0.1 mM CaCl₂, 1 mM DTT, 0.02% NaN₃).

Cleavage of the GST tag left two amino acids from the PreScission protease recognition site. In addition, cloning the fascin DNA into the multiple cloning site introduced three extra amino acids, resulting in a GPLGS N-terminal extension. The molecular weight (MW) of two independent preparations of recombinant fascin-1 was determined by mass spectrometry (Environmental Laboratory of Mass Spectrometry, Department of Biophysics, Institute of Biochemistry and Biophysics, Warsaw, Poland) as 54,926 Da and 54,923 Da, which is in good agreement with the theoretical MW (54,919 Da) of mouse fascin-1 with the five–amino acid N-terminal extension. Protein concentration was determined spectrophotometrically at 280 nm using a molar extinction coefficient of 66,350 M^−1^ × cm^−1^, calculated in ExPASy (https://web.expasy.org/protparam/) based on the amino acid sequence and assuming all Cys residues were reduced.

### Actin preparation

Actin was isolated from New Zealand rabbit back and hind leg muscle. Fresh meat was a gift from the Department of Pathobiochemistry and Clinical Chemistry, Collegium Medicum in Bydgoszcz. Procedures of the Committee for Ethical Experiments on Animals of Collegium Medicum were used to sacrifice the animals. The protein was purified according to [50] as described in [51].

### Co-sedimentation assay to analyze tropomyosin and fascin binding to F-actin

Binding of fascin and Tpm2 isoforms to single filaments or bundles was assessed by centrifugation. High-speed centrifugation (HSC) was performed for 1 h at 40,000 rpm using the Beckman 42.2Ti rotor at 20 °C to analyze binding to both single and bundled actin filaments. Binding specifically to actin bundles was evaluated by low-speed centrifugation (LSC) for 30 min at 9,000 rpm using the same rotor. Prior to each experiment, G-actin was polymerized for 30 min in 100 mM NaCl and 2 mM MgCl₂. The assay buffer contained 5 mM Tris–HCl, 100 mM NaCl, 2 mM MgCl₂, and 1 mM DTT, pH 7.5.

Binding of Tpm2 isoforms to either 3.0 µM F-actin or to actin bundles (pre-formed by incubating 3.0 µM F-actin with 0.3 µM fascin for 20 min before the experiment) was assessed by titration with Tpm2 isoforms at concentrations ranging from 0 to 3.0 µM.

Binding of fascin to 3.0 µM F-actin was assessed by incubating the filaments with increasing concentrations of fascin (0 to 1.1 µM) for 1 h at room temperature (RT). Where indicated, Tpm was pre-incubated with F-actin at a saturating concentration of 2.0 µM for 15 min prior to the addition of fascin. To check Tpm-mediated inhibition of fascin binding, Tpm was added at concentrations ranging from 0 to 3.0 µM and incubated with F-actin for 15 min before the addition of 0.3 µM fascin.

Proteins in the pellets and in the supernatants were separated by SDS-PAGE on 12% gels. Protein bands were quantified using Image Lab software (Bio-Rad Inc., Hercules, CA, USA). To normalize the data, densitometric Tpm/actin or fascin/actin ratios were divided by the maximum ratio observed in each experiment. Normalized values were plotted against the concentration of unbound Tpm or fascin.

The experimental points were fitted to the Hill equation to obtain the apparent binding constant K_app_ and the Hill cooperativity coefficient α^H^:

$$v=\frac{{{n\times L}^{\alpha }}^{H}\times {{{K}_{app}}^{\alpha }}^{H}}{{{1+L}^{\alpha }}^{H}\times {{{K}_{app}}^{\alpha }}^{H}}$$

where: $v$ represents the fractional saturation of F-actin with Tpm or fascin; n is the maximal binding capacity of the actin filament for tropomyosin or fascin; L denotes the concentration of unbound tropomyosin or fascin; K_app_ is the apparent association constant; α^H^ is the Hill cooperativity coefficient. Values for K_app,_ α^H^ and their standard errors, reflecting the deviation of the fitted curve from the experimental data, were obtained from the nonlinear regression analysis performed in SigmaPlot 12.5 for each binding curve.

Dissociation of fascin from F-actin-fascin complex by Tpm2 isoforms or inhibition of fascin binding to F-actin–Tpm complexes, was assessed by densitometric analysis of fascin band intensities in the pellet fractions on SDS-PAGE gels using Image Lab (Bio-Rad, Inc., Hercules, CA, USA). Band intensities were normalized by dividing each value by the fascin band intensity in the absence of Tpm, and the resulting values were plotted against the total Tpm concentration. Experimental data were fitted to an exponential decay model.

### F-actin bundling assay

Actin-bundling activity of fascin in the absence and presence of Tpm2 isoforms was assessed by LSC assay as described in [52]. Briefly, 5 µM G-actin was polymerized at RT for 30 min with 2 mM MgCl_2_ and 100 mM NaCl. If present, tropomyosins were added to a final concentration of 3.33 µM and incubated for an additional 30 min before titration with increasing concentrations of fascin (0–1.11 µM). Bundling reactions were allowed to proceed for 1 h, followed by centrifugation at 9,000 rpm in the Beckman Optima L-90 K ultracentrifuge, rotor 42.2Ti, for 30 min at 20 °C. Pellets and supernatants were analyzed by SDS-PAGE on 12% polyacrylamide gels. The relative actin-bundling activity of fascin was calculated using the following formula:

$${R}_{B}=\frac{P-{P}_{0}}{P+S-{P}_{0}}$$

where: R_B_ represents the relative bundling activity; P densitometric intensity of the actin band in the pellet at a given fascin concentration; P_0_ is the intensity of the actin band in the pellet in the absence of fascin; and S is the intensity of the actin band in the corresponding supernatant.

### Pull-down assay

Direct binding of Tpms to fascin was analyzed using a pull-down assay. Bacterial lysate prepared from *E. coli* Rosetta cells, containing GST-tagged fascin-1, was incubated with glutathione–agarose resin overnight at 4 °C on a rotating wheel to immobilize fascin-1. The resin was then divided into 200 µL aliquots, extensively washed (10–11 times with 400 μL of G-buffer and 2 times with digestion buffer) and incubated with 200 µL of 30 µM Tpm isoforms in binding buffer (5 mM Tris–HCl, pH 7.5, 100 mM NaCl, 2 mM MgCl₂, 1 mM DTT, 0.02% NaN₃) overnight at 4 °C with rotation. The 30 µM concentration of Tpm used for incubation with fascin-loaded resin was selected empirically to allow robust detection of all three Tpm isoforms on SDS-PAGE, high enough for binding yet below the threshold for non-specific aggregation or resin overloading. Following incubation, the resin was washed thoroughly with the above buffer adjusted to 30 mM NaCl. This washing step was designed to reduce the affinity between Tpm and fascin, thereby highlighting differences in binding among Tpm isoforms, and to minimize nonspecific interactions that could occur at physiological ionic strength.

Bound proteins were eluted using PreScission Protease, which specifically cleaves the GST tag. Control samples were prepared in parallel, omitting GST–fascin during resin loading. All eluates were resolved by SDS-PAGE and analyzed for Tpm isoform retention.

### Fluorescent microscopy analysis of fascin-mediated actin bundle formation

Fascin-mediated actin filament bundling was directly visualized using an Olympus IX83 inverted fluorescence microscope. Actin filaments were fluorescently labeled with tetramethylrhodamine cadaverine (TRC; Zedira, Darmstadt, Germany) using bacterial transglutaminase, as previously described [53].

Before microscopic analysis, TRC-labeled G-actin was polymerized in F-buffer (100 mM KCl, 4 mM MgCl₂, 20 mM Tris, pH 7.5) overnight at 4 °C. The resulting TRC–F-actin (120 nM) was used for bundling either without Tpm or was preincubated with Tpm (80 nM) for 30 min. To form bundles, the filaments were incubated with fascin (120 nM) overnight at 4 °C. Bundles were visualized in a flow chamber assembled from two coverslips (24 × 24 mm and 32 × 24 mm) joined with parafilm spacers and coated with positively charged lipids: DPPC (1,2-dipalmitoyl-sn-glycero-3-phosphocholine; Sigma-Aldrich, St Louis, MO, USA) and TAP (1,2-dipalmitoyl-3-trimethylammonium-propane; Sigma Aldrich), at a DPPC:TAP weight ratio of 9:1 [54]. Fascin-bound TRC–F-actin filaments, with or without Tpm, were introduced into flow cells freshly rinsed with blocking buffer (20 mM Tris–HCl, pH 7.5, 100 mM KCl, 4 mM MgCl₂, and 5 mg/mL BSA). Unbound filaments were removed using buffer supplemented with the following antioxidant enzymes: 0.1 mg/mL of glucose oxidase (Sigma Aldrich), 0.01 mg/mL of catalase (Sigma Aldrich), and 3 mg/mL of glucose (Sigma Aldrich). Randomly selected fields (100 × magnification) were imaged. Fluorescence intensity of single filaments and bundle cross-sections was analyzed using the Line Profile tool in cellSens Dimension software (Olympus Life Science, Tokyo, Japan). The number of filaments per bundle was calculated by dividing the fluorescence intensity of the bundle cross-section by that of individual filaments. Bundle length and width were measured using the Measurement tool in cellSens Dimension.

### Plasmid construction

To generate the plasmids pEGFP-C1-TPM2.1, pEGFP-C1-TPM2.3 and pEGFP-C1-TPM2.4, RNA was isolated from HOS osteosarcoma cells using GenElute™ Mammalian Total RNA Miniprep Kit (Sigma-Aldrich) and reverse transcribed using QuantiTect® Reverse Transcription Kit (Qiagen, Hilden, Germany). *TPM2.1*, *TPM2.3* and *TPM2.4* gene isoforms were subsequently amplified by PCR (primer sequences in Table 1) and cloned into KpnI/BamHI sites of plasmid pEGFP-C1. All plasmid sequences were verified by Sanger sequencing.

**Table 1:** Primer sequences

*TPM2.1*	GAGCGGTACCGCCATGGACGCCATCAAGAAGAAGATGC
	GAGCGGATCCTCACAGGTTGTTGAGTTCCAG
*TPM2.3*	GAGCGGTACCGCCATGGACGCCATCAAGAAGAAGATGC
	GAGCGGATCCTCACAGGTTGTTGAGTTCCAG
*TPM2.*4	GAGCGGTACCGCCATGGACGCCATCAAGAAGAAGATGC
	GAGCGGATCCTCAGAGGGAGGTGATGTCATTGAG
*TPM2 qPCR*	CCAGGAGAAACTGAAGCAGG
	CTCCTCATCCTTCATGGCCC
*FSCN-1 qPCR*	GTGGAGACAGCAGGGGACTC
	TAGGCGCCATCGTTGAACTC
*ACTB qPCR*	GATTCCTATGTGGGCGACGA
	AGGTCTCAAACATGATCTGGGT
*GAPDH qPCR*	TCGGAGTCAACGGATTTGGT
	TTCCCGTTCTCAGCCTTGAC

### Cell cultures

Osteosarcoma cell line SAOS-2 and SAOS-2 LM5 cells were kindly provided by Bruno Fuchs. SAOS-2 LM5 cells, a highly metastatic variant of parental SAOS-2 cells, was derived previously by repeated in vivo selection of metastatic cells in mice [55]. Both cell lines were authenticated using short tandem repeat profiling by Generi Biotech s.r.o. Cells were cultured in a humidified incubator (37 °C, 5% CO_2_) in DMEM (Sigma-Aldrich) with 10% fetal bovine serum (FBS) (Invitrogen, Carlsbad, CA, USA), 2 mM L’-glutamine, 100 U/ml penicillin and 100 μg/ml streptomycin (Lonza, Basel, Switzerland).

### RNA isolation, cDNA synthesis and quantitative PCR (qPCR)

8 × 105 SAOS-2 LM5 cells were cultured in 10 ml Petri dish for 48 h, transfected with pEGFP-C1-TPM2.1, pEGFP-C1-TPM2.3, pEGFP-C1-TPM2.4, or mock-transfected using Lipofectamine® LTX (Invitrogen) and harvested 24 h later. Total RNA was isolated using GenElute™ Mammalian Total RNA Miniprep Kit (Sigma-Aldrich) and cDNA was synthesized using QuantiTect® Reverse Transcription Kit (Qiagen, Hilden, Germany). qPCR was performed with KAPA SYBR Fast Master mix (KAPA Biosystems, Wilmington, MA) with primers using the LightCycler 480 (Roche, Basel, Switzerland). GAPDH was used as an internal control. The qPCR data were analyzed by the 2-ΔCt method. All primer sequences are shown in Table 1.

### Immunoblotting

1 × 106 SAOS-2 and SAOS-2 LM5 cells were cultured in 10 ml Petri dish for 48 h. Alternatively, 4 × 105 SAOS-2 LM5 cells were cultured in 5 ml Petri dish for 48 h, transfected with pEGFP-C1-TPM2.1, pEGFP-C1-TPM2.3, pEGFP-C1-TPM2.4, or mock-transfected using Lipofectamine® LTX (Invitrogen) and harvested 24 h later. Cells were lysed and proteins resolved by SDS-PAGE and immunoblotted as described previously [56]. Blots were probed with anti-Tpm2 (11,038–1-AP, Proteintech, Rosemont, IL), anti-fascin (54,545, Cell Signaling Technology, Danvers, MA), anti-β-actin (4970, Cell Signaling Technology) or anti-α-tubulin (T9026, Sigma-Aldrich) antibodies and subsequently with horseradish peroxidase-conjugated anti-mouse or anti-rabbit secondary antibodies (Sigma-Aldrich). Signal was obtained with a standard ECL procedure using Clarity™ Western ECL Substrate (Bio-Rad, Inc., Hercules, CA, USA).

### Immunofluorescence staining and co-localization analysis

SAOS-2 LM5 cells were seeded on sterile glass coverslips in a 5 ml Petri dish. The next day, the cells were transfected with pEGFP-C1-TPM2.1, pEGFP-C1-TPM2.3, pEGFP-C1-TPM2.4, or mock-transfected using Lipofectamine® LTX (Invitrogen). After 48 h, the coverslips were washed with 1 × PBS and cells were fixed with 4% PFA in 1 × PBS for 10 min at RT. Then, the coverslips were incubated with 5% FBS in 1 × PBS for 1 h at RT. Cells were then incubated with anti-fascin antibody (ab126772, Abcam, Cambridge, UK, dilution 1:200) in 1 × PBS with 1% BSA and 0.3% Triton**™** X-100 (Sigma-Aldrich) at 4 °C overnight. The coverslips were washed three times in 1 × PBS and subsequently incubated with Goat anti-Rabbit IgG (H + L) Highly Cross-Adsorbed Secondary Antibody, Alexa Fluor™ 546 (Invitrogen, dilution 1:500) in 1 × PBS with 1% BSA and 0.3% Triton™ X-100 for 1 h at RT. Then, the coverslips were washed in 1 × PBS and incubated with Alexa Fluor™ 647 Phalloidin (Invitrogen) for 20 min at RT. The coverslips were then mounted with ProLong Gold Antifade Mountant with DAPI (Thermo Fisher Scientific, Waltham, MA, USA) and visualized by fluorescence microscopy.

Confocal images were acquired with a Laser scanning confocal microscope Zeiss LSM 880 with AiryscanFast module (Carl Zeiss, Oberkorchen, Germany) using Plan-Apochromat, Oil 63 ×/1.40 objective. Argon 488 nm laser, 561 nm solid-state, and 633 nm solid-state lasers were employed. GFP was excited at 488 nm and emitted light was detected at 524 nm, AlexaFluor^®^ 546 was excited at 561 nm and detected at 601 nm, and Alexa Fluor^®^ 647 was excited at 633 nm and detected at 697 nm. Airyscan images were processed using ZEN (black edition) software (Carl Zeiss).

Co-localization analysis was conducted using the ZEN Blue Software (Carl Zeiss). The co-localization of fascin and F-actin was quantified by calculating the Co-localization Coefficient (Coloc. Coeff. 2) which defined by the following equation:

$$Coloc. Coeff. 2= \frac{\#{pixels}_{Ch2 colocalised}}{\#{pixels}_{Ch2 total}}$$

The coefficient represents the pixel-to-pixel co-localization of channel 2 (F-actin) to channel 1 (fascin). This strategy was selected to allow quantification of fascin-free F-actin in cells. The coefficients are expressed as values ranging from 0.0 to 1.0. Thresholds were carefully adjusted to exclude background noise and ensure the sensitive detection of signals in each channel. For cells overexpressing Tpm2 isoforms, only cells with confirmed overexpression (GFP-positive cells) were included in the analysis. From each image, multiple z-stacks were selected and analyzed. The data from the z-stacks within a single image were averaged to ensure an accurate and consistent assessment of three-dimensional cellular features. A total of 65 mock-transfected cells, 29 Tpm2.1-expressing cells, 29 Tpm2.3-expressing cells, and 28 Tpm2.4-expressing cells were analyzed to ensure comprehensive data acquisition. Similarly, co-localization between Tpm2 and F-actin was analyzed in Tpm2-overexpressing cells.

### Cell morphology analysis

A total of 4 × 10^5^ SAOS-2 LM5 cells were cultured in 5 ml Petri dishes for 48 h and transfected with pEGFP-C1-TPM2.1, pEGFP-C1-TPM2.3, pEGFP-C1-TPM2.4, or mock-transfected using Lipofectamine® LTX (Invitrogen). Twenty-four hours post-transfection, cells were trypsinized and 20,000 cells were seeded into each well of a 96-well plate and incubated overnight under standard culture conditions. Live-cell imaging was performed the following day using the Incucyte^®^ S3 Live-Cell Analysis System (Sartorius, Göttingen, Germany). The acquisition settings were as follows: Scan type – Standard; Analysis mode – Adherent Cell-by-Cell; Imaging channels – Phase contrast and Green fluorescence (acquisition time: 300 ms); Objective – 20 ×; Images per well – 4. Images were exported with scale bars using the Incucyte^®^ Live Cell Analysis Software and further analyzed in ImageJ. For morphological evaluation, each cell was manually outlined using the freehand selection tool. Cell area and circularity were quantified using the Measure function. For each condition, a minimum of 400 cells was analyzed.

## Results

### Actin-binding properties of acetylated recombinant Tpm2 isoforms

The majority of Tpm2.1 expressed in cells is N-terminally acetylated [25], a modification that is crucial for actin affinity and the regulatory functions of Tpm [57–59]. However, bacterially produced recombinant proteins are not acetylated due to the absence of N-terminal acetylase in prokaryotic cells. To obtain acetylated human cytoskeletal Tpm2 isoforms, the *E.coli* expression strain BL21(DE3) was co-transformed with pET plasmid containing DNA sequences of individual Tpm2 isoforms and plasmid carrying DNA of the NatB complex to enable acetylation [60]. Mass spectrometry confirmed the molecular mass of the isolated and purified proteins, verifying N-terminal acetylation in over 90% of the protein population.

**Fig. 2:**
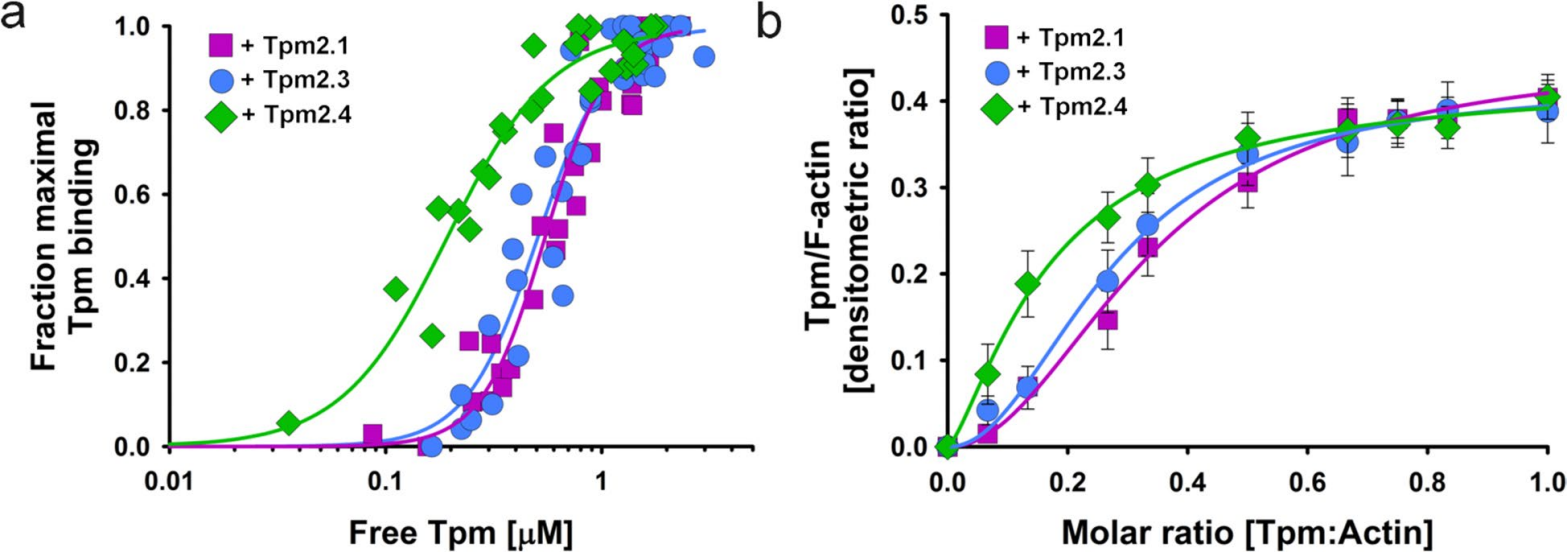
Binding of Tpm2 isoforms to F-actin obtained by a co-sedimentation assay at HSC as a function of free (**a**) and total (**b**) concentrations of tropomyosins. Conditions: 5 mM Tris, 2 mM MgCl_2_, 100 mM NaCl, 1 mM DTT, pH 7.5. Protein concentration: 3.0 μM F-actin, Tpm2 isoforms at 0–3.0 μM

To determine whether sequence differences between Tpm2 isoforms (Fig. 1) affect their interactions with actin filaments, we compared the actin-binding parameters of Tpm2.1, Tpm2.3, and Tpm2.4 using a high-speed centrifugation (HSC) co-sedimentation assay. The binding curves in Fig. 2 show that Tpm2.4 binds to F-actin with the highest affinity, while the affinities of Tpm2.1 and Tpm2.3 are lower and do not significantly differ from each other (Table 2). Although Tpm2.4 appears to bind with lower cooperativity, the difference in Hill coefficients between Tpm2.4 and the other isoforms was not statistically significant (Table 2).

**Table 2:** Parameters of Tpm2 isoforms binding to F-actin

	− fascin			+ fascin		
	Tpm2.1	Tpm2.3	Tpm2.4	Tpm2.1	Tpm2.3	Tpm2.4
K_app_ (μM^−1^)	1.8 ± 0.1	1.9 ± 0.1	5.1 ± 0.3*	1.5 ± 0.4 *1.5* ± *0.2*	0.8 ± 0.4 *2.0* ± *0.2*	5.2 ± 0.3* *5.7* ± 0.9*
α^H^	2.9 ± 0.4	2.6 ± 0.4	1.8 ± 0.3	1.9 ± 0.1 *2.0* ± *0.3*	1.2 ± 0.1 *1.9* ± *0.3*	0.9 ± 0.3 *0.9* ± *0.2*

Conditions as in the captions to Fig. 2 and Fig. 5. The K_app_ values in regular font were obtained by HSC, while those in itallics were obtained by LSC. *statistically significant difference between Tpm2.4 and two other Tpm2 isoforms (p ≤ 0.05, one-way ANOVA). The data are from 3–4 independent experiments

Analysis of F-actin saturation as a function of total Tpm2 concentration revealed that all Tpm2 isoforms fully saturated actin filaments at a 1:1.5 Tpm:actin molar ratio (Fig. 2b). This ratio was therefore used in subsequent experiments to examine the regulatory properties of the Tpm2 isoforms.

### Effects of Tpm2 isoforms on bundling of F-actin by fascin-1

**Fig. 3:**
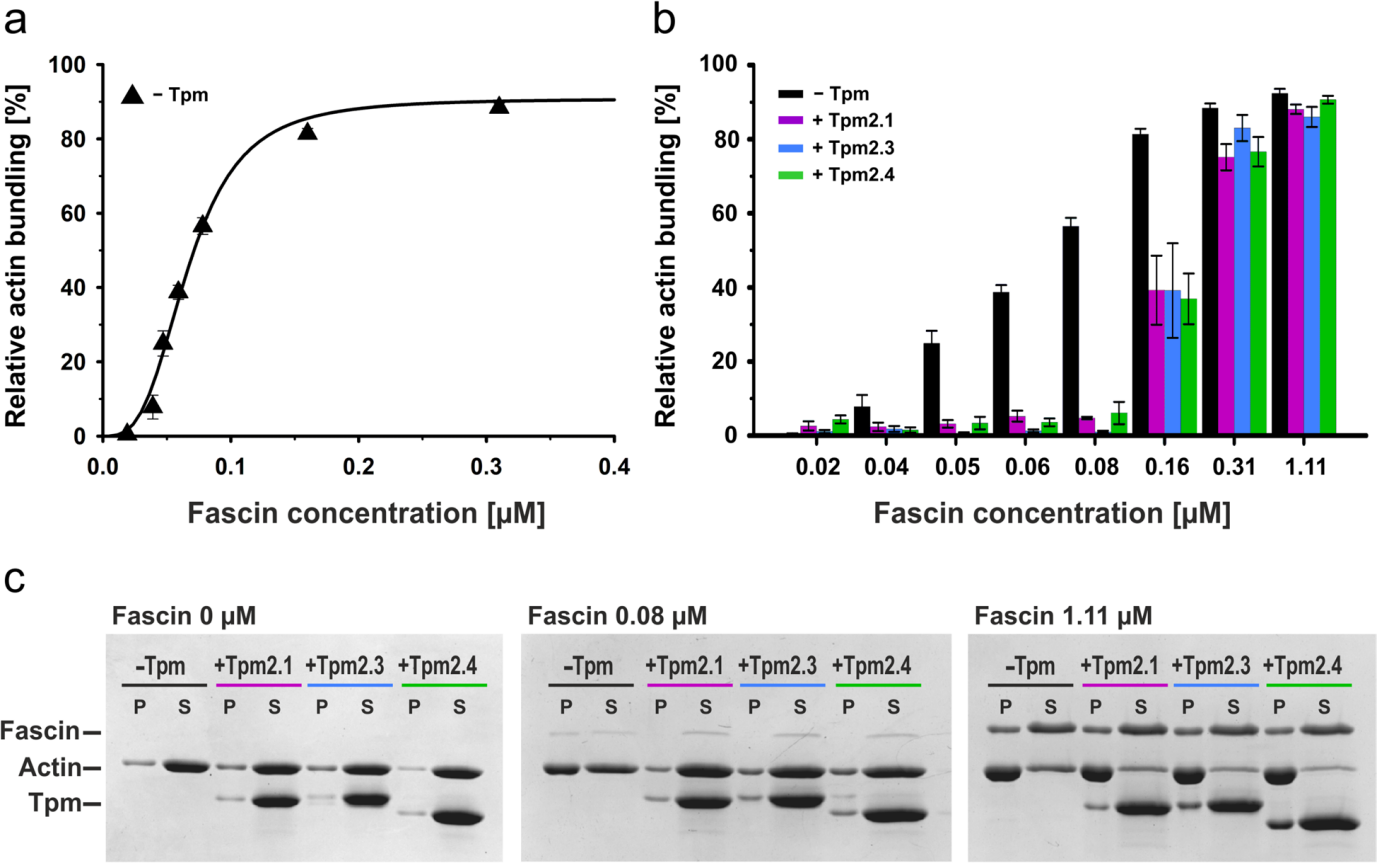
Bundling activity of fascin obtained by LSC in the absence (**a**) and presence (**b**) of Tpm2 isoforms. **c** Representative pellet (P) and supernatant (S) fractions at given fascin concentrations analyzed by SDS-gel electrophoresis. Conditions: 5 mM Tris–HCl, 2 mM MgCl_2_, 100 mM NaCl, 1 mM DTT, pH 7.5. Protein concentrations: 5 μM F-actin, 0–1.11 μM fascin, 3.3 μM Tpm2 isoforms (1:1.5 Tpm:actin molar ratio). Data in panels **a** and **b** are averaged from 3 to 7 independent experiments

The bundling activity of fascin was analyzed by LSC (9,000 rpm). Under these conditions, neither fascin nor single actin filaments sediment, allowing their separation from higher-order actin structures, such as bundles [6, 11]. F-actin was incubated with fascin at concentrations ranging from 0.019 to 1.11 μM, corresponding to fascin:actin molar ratio from 1:263 to 1:4.5. The relative actin bundling curve shown in Fig. 3a confirmed that, as previously reported [6], the process is cooperative. Approximately 90% of F-actin was bundled, with the maximal effect achieved at about 0.3 μM fascin (1:16 fascin:actin molar ratio).

To assess the ability of Tpm2 isoforms to regulate fascin-mediated bundling, actin filaments were first saturated with Tpm2 isoforms and then incubated with fascin to form bundles. As shown in Fig. 3b, all Tpm2 isoforms inhibited bundling at low fascin concentrations. This strong inhibitory effect was evident at fascin:actin molar ratios ranging from 1:263 to 1:64, where bundling of actin in the absence of Tpm2 increased from 0 to approximately 55%. The inhibition was partially relieved at high fascin concentrations, with bundling comparable to that of unregulated actin achieved only at a fascin concentration approximately three times higher than that required for maximal bundling of unregulated actin. Despite differences in the regulation of fascin binding to actin, we observed no significant differences among Tpm2 isoforms in their ability to regulate fascin’s actin-bundling activity. This might be due to variability in the experimental data from the bundling assay performed using the LSC method.

As shown by the SDS-gels of pellets obtained during LSC (Fig. 3c), in the presence of Tpm2 the intensity of the actin bands remains unchanged up to a 0.08 μM fascin (1:64 fascin:actin molar ratio), compared to the absence of fascin. This indicates that at these fascin levels, Tpm2 isoforms fully inhibit bundling. Actin bundles obtained at high fascin:actin ratios contained fascin and Tpm2 isoforms, indicating that both proteins can coexist on the actin filament. The requirement for high fascin concentrations for efficient bundling may suggest that the presence of Tpm alters fascin’s affinity for actin.

### Effects of Tpm2 isoforms on interactions of fascin-1 with actin filaments

**Fig. 4:**
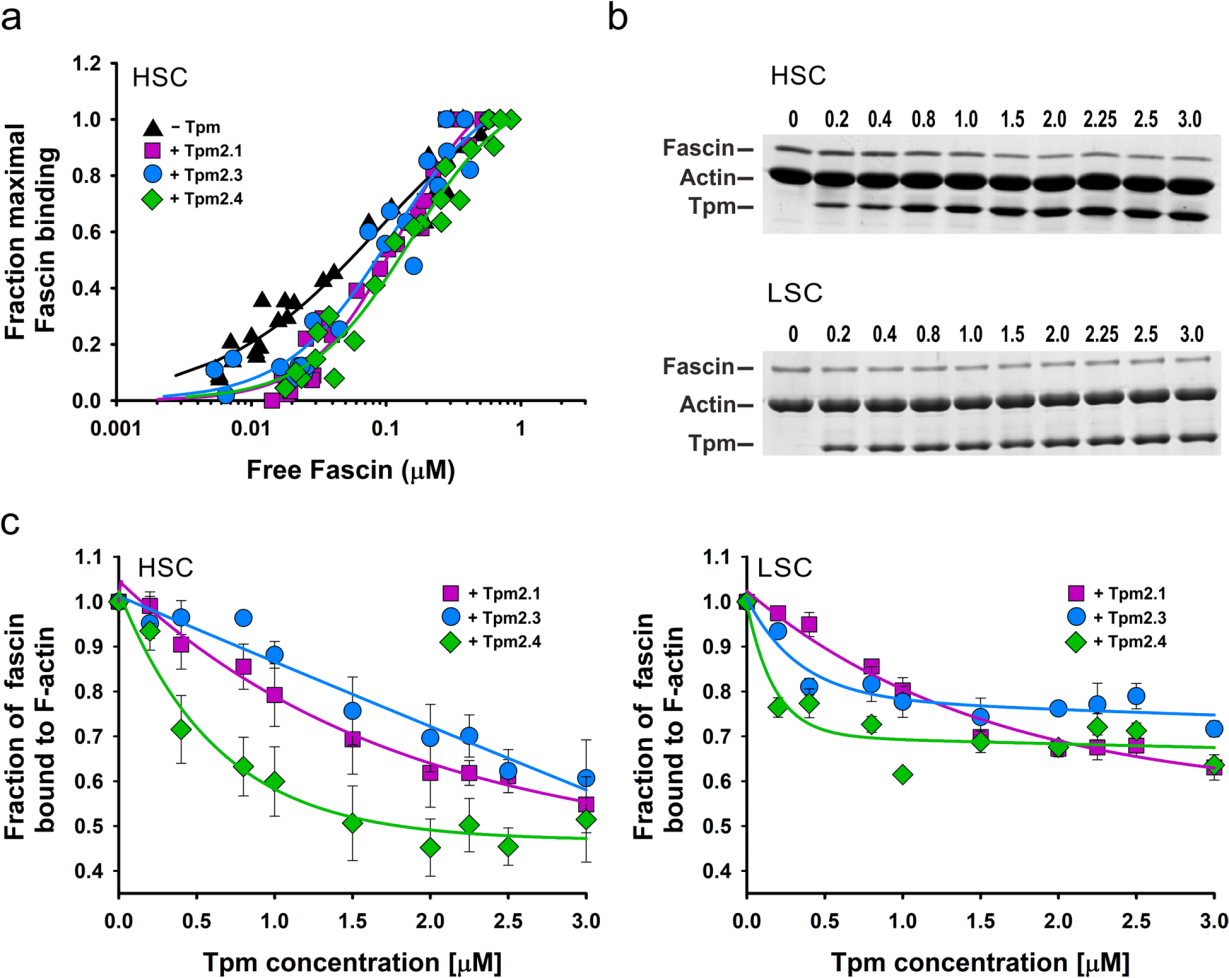
Effects of Tpm2 isoforms on fascin binding to F-actin. **a** Binding of fascin to F-actin. **b** Representative SDS-PAGE of pellets obtained following HSC and LSC in the presence of increasing concentrations of Tpm2.4. **c** Partial inhibition of fascin binding to actin by Tpm2 isoforms. Conditions: 5 mM Tris–HCl, 2 mM MgCl_2_, 100 mM NaCl, 1 mM DTT, pH 7.5. Protein concentrations were: 3.0 μM F-actin, ± 2 μM Tpm2 isoforms, and 0–1.1 μM fascin (**a**); 3.0 μM F-actin with Tpm2 isoforms added at 0–3.0 μM followed by 0.3 μM fascin (**b** and **c**)

To assess whether coating actin filaments with Tpm2 isoforms affects the actin affinity of fascin, we compared fascin binding to F-actin alone with F-actin saturated with Tpm2 isoforms. Binding of fascin to actin was analyzed by co-sedimentation of F-actin with increasing concentrations of fascin using HSC, without distinguishing between its association with individual filaments and filament bundles. The binding constant indicates that fascin alone strongly binds to F-actin (Table 3), which is in good agreement with previous data obtained with skeletal Tpm isoform [61]. Notably, only Tpm2.4 significantly reduced this affinity (Fig. 4a). However, all Tpm2 isoforms significantly increased the cooperativity of fascin’s interaction with actin (Table 3).

**Table 3:** Parameters of fascin-1 binding to actin in the absence and presence of Tpm2 isoforms

	K_app_ (μM^−1^)	α^H^
F-actin	11.5 ± 4.5	0.7 ± 0.1
F-actin + Tpm2.1	8.0 ± 1.5	1.3 ± 0.1*
F-actin + Tpm2.3	9.4 ± 2.6	1.1 ± 0.2*
F-actin + Tpm2.4	6.7 ± 1.3*	1.2 ± 0.1*

Conditions as in the caption to Fig. 4.*statistically significant difference between binding and cooperativity in the presence and absence of Tpm2 carried out by one-way ANOVA. Data averaged from 3 independent experiments

The observed decrease in affinity in the presence of Tpm2 isoforms may result from competition caused by the high Tpm concentrations used to fully saturate the filaments (2 μM). To verify whether Tpm2 isoforms are capable of inhibition of fascin binding to actin, we analyzed fascin binding to F-actin which was incubated at increasing Tpm2 concentrations prior to adding fascin at the saturating, 0.3 μM concentration (1:16 fascin:actin molar ratio). The protein content of pellets collected by HSC and LSC (Fig. 4b) was then analyzed. The results indicate that the amount of fascin bound to F-actin decreased with increasing concentrations of Tpm2, although the inhibition was only partial (Fig. 4c). Maximal inhibition of fascin binding to both single and bundled filaments was observed at approximately 1.5 µM Tpm2.4, the concentration required to fully saturate F-actin with this isoform. Consistent with their lower affinities for actin, Tpm2.1 and Tpm2.3 were less effective at blocking fascin binding.

### Binding of Tpm isoforms to fascin-bundled actin

**Fig. 5:**
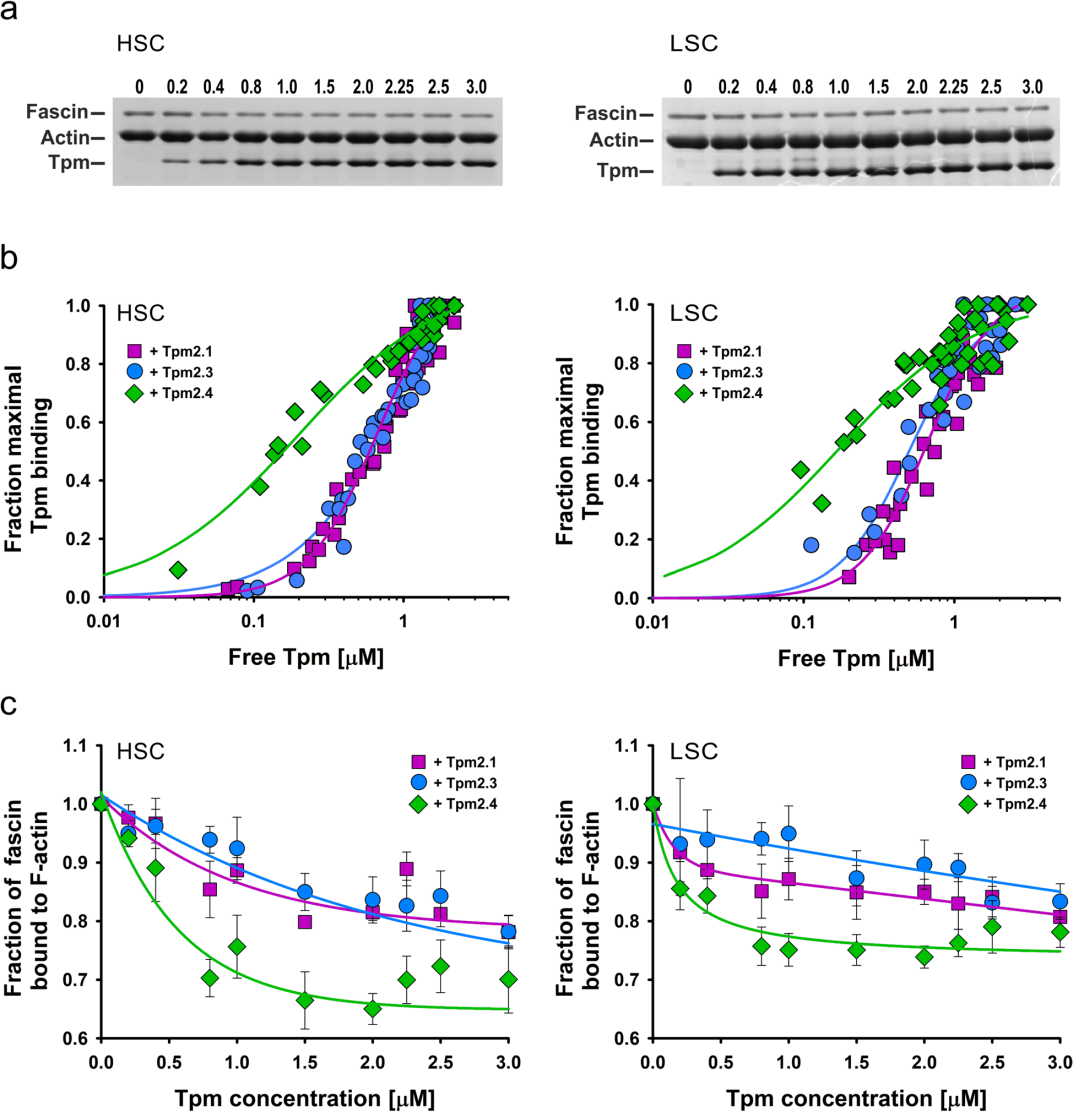
Binding of Tpm2 isoforms to fascin-bundled F-actin assessed by co-sedimentation at HSC (left) and LSC (right).** a** Representative SDS-PAGE of pellets obtained following HSC and LSC in the presence of increasing micromolar concentrations of Tpm2.4, as indicated above each lane. **b** Actin-binding curves of Tpm2 isoforms. **c** Partial dissociation of fascin from F-actin caused by Tpm binding. Conditions: 5 mM Tris, pH 7.5, 2 mM MgCl_2_, 100 mM NaCl, 1 mM DTT, 3.0 μM F-actin incubated with 0.3 μM fascin was titrated with Tpm2 isoforms at 0 to 3 μM. The data in **b** and **c** were from 3 or 4 independent experiments

To assess whether fascin had a reciprocal effect on Tpm2's interaction with actin, we analyzed the binding of Tpm2 to actin in the presence of fascin, added to actin at a 1:16 molar ratio (Fig. 5a). In contrast to the results illustrated in Fig. 4, in this experimental variant fascin was bound to actin prior to Tpm2. The binding curves shown in Fig. 5b demonstrate that in the presence of fascin, Tpm2.4 exhibited the highest affinity for actin, while Tpm2.1 and Tpm2.3 bound with lower affinity. No significant difference in affinity between these two isoforms was detected. The binding curves illustrating fractional Tpm2 binding to total actin (HSC) and actin bundles (LSC) are very similar. The K_app_ values obtained in the presence of fascin by LSC (Table 2, italics) were not significantly different from those obtained by HSC, indicating the same affinity of Tpm2 isoforms. Thus, fascin does not appear to have an effect on the binding of Tpm2 isoforms to actin filaments.

SDS-gels showing protein composition in pellets collected by HSC and LSC (Fig. 5a) revealed that increasing actin saturation with Tpm2 caused partial dissociation of fascin from the filaments. Quantitative comparisons of all Tpm2 isoforms, shown in Fig. 5c, demonstrated that fascin was removed from the filaments most efficiently by Tpm2.4. The amount of bound fascin was reduced by approximately 30%. Tpm2.1 and Tpm2.3 required higher concentrations to achieve maximal effect. Since the processes analyzed by LSC produced similar fascin-dissociation curves, it appears that Tpm2 isoforms optimize the protein composition of bundles.

### Direct interactions between fascin-1 and Tpm2 isoforms

Although fascin’s affinity for actin filaments was reduced by Tpm2 isoforms, it was not entirely displaced from the filaments even at high Tpm concentrations. This suggests that the regulation of fascin-actin interactions by Tpm2 is more complex than only steric blocking of binding sites on actin. Another mechanism of regulation can involve direct interactions between fascin and Tpm.

**Fig. 6:**
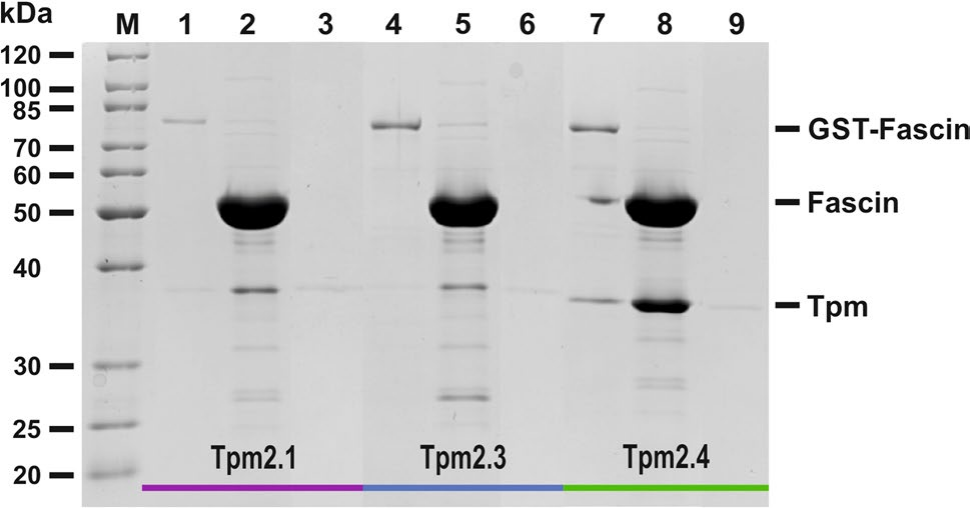
Direct interactions between fascin and Tpm2 isoforms. Lanes: M- Precision plus protein standard (Bio-Rad, Inc., Hercules, CA, USA); 1, 4, 7 – last wash after incubation of tropomyosin isoform with GST-fascin bound to glutathione–agarose resin; 2, 5, 8 – eluted complex of fascin with tropomyosin isoforms after cutting GST with PreScission Protease; 3, 6, 9 – controls of non-specific binding of Tpm2 isoforms to the resin in the absence of GST-fascin

To test this possibility, we performed pull-down experiments in which Tpm2 isoforms were incubated with the GST-tagged fascin immobilized on glutathione–agarose. The fascin-Tpm2 complexes were released by proteolysis of the GST-tag and analyzed electrophoretically. The results presented in Fig. 6 demonstrate that, under these conditions, all Tpm2 isoforms were capable of interacting with fascin. However, the relatively low intensities of the Tpm2.1 and Tpm2.3 bands suggest that their affinities for fascin were low. By contrast, Tpm2.4 exhibited substantially higher affinity, as indicated by an approximately fivefold increase in band intensity.

### Effects of Tpm2 isoforms on the architecture of the fascin–actin bundles

**Fig. 7:**
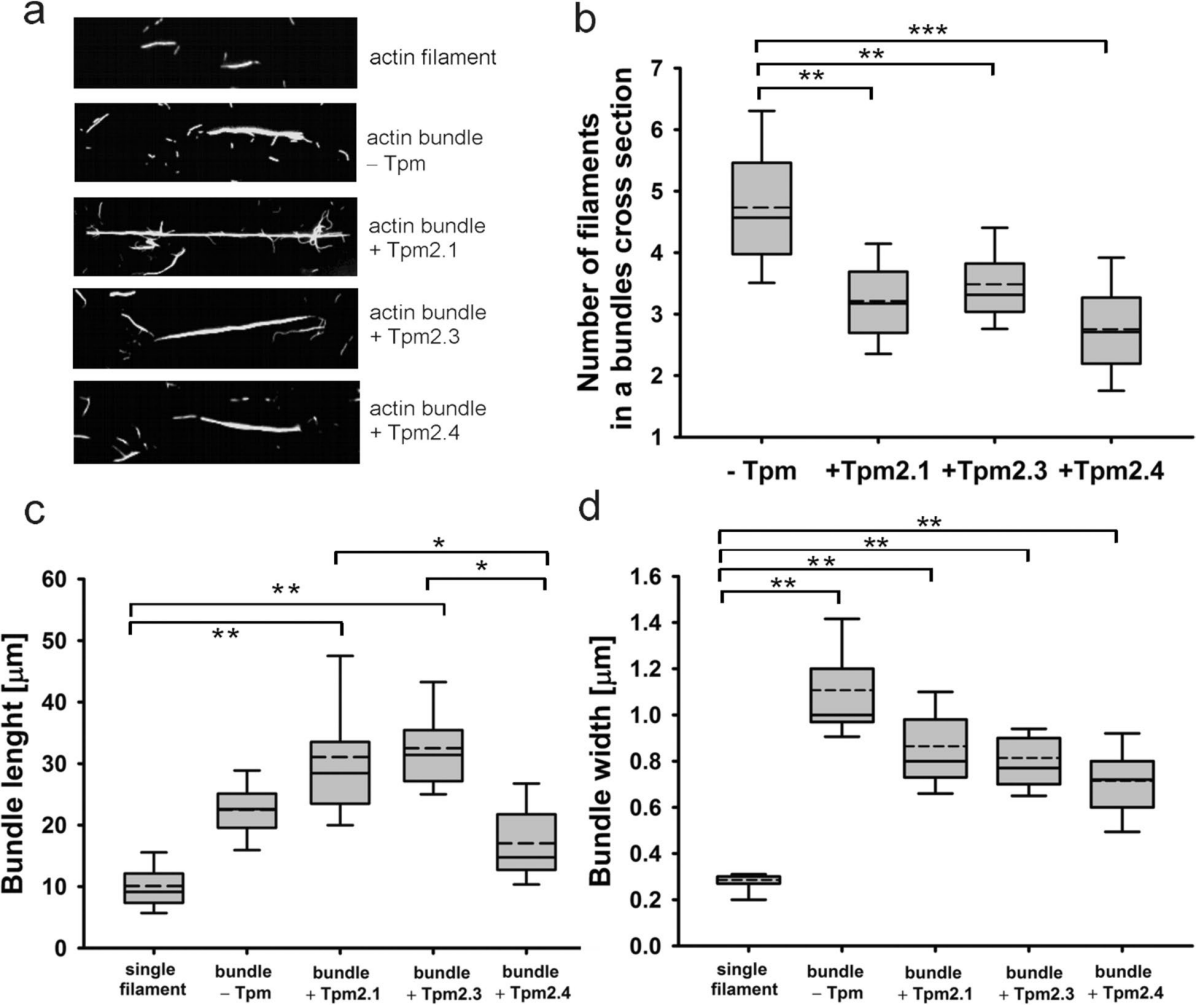
Regulation of actin–fascin bundle size by Tpm2 isoforms. **a** Representative images of individual actin filament and actin bundles formed in the absence or presence of Tpm2 isoforms. Quantification of the number of filaments per bundle (**b**), bundle length (**c**), and bundle width (**d**). Graphs display the median (solid line) and mean (dashed line), with error bars indicating variability. Data represent averages from 50 to 70 actin bundles per condition. Statistical significance was assessed by one-way ANOVA (*p ≤ 0.05, **p ≤ 0.01, ***p ≤ 0.001)

The ability of Tpm2 isoforms to regulate bundle length, width, and the number of actin filaments per bundle was assessed by fluorescence microscopy image analysis. Based on the fluorescence intensity of cross-sections of individual filaments and bundles (Fig. 7a), the fascin–actin bundles were estimated to contain between 3 and 8 filaments, with an average of 4.68 ± 0.19 filaments per bundle. Binding of Tpm2 isoforms to actin filaments prior to fascin-induced bundling significantly reduced bundle thickness, with Tpm2.4 having the most pronounced effect, decreasing the number of filaments per bundle to 2.80 ± 0.32 (Fig. 7b). Interestingly, the Tpm2 isoforms exerted distinct effects on bundle length. Whereas Tpm2.1 and Tpm2.3 increased the average bundle length, Tpm2.4 had no significant effect compared with actin bundles formed in the absence of Tpm2 (Fig. 7c).

### Expression of Tpm2 and fascin in osteosarcoma cell lines

**Fig. 8:**
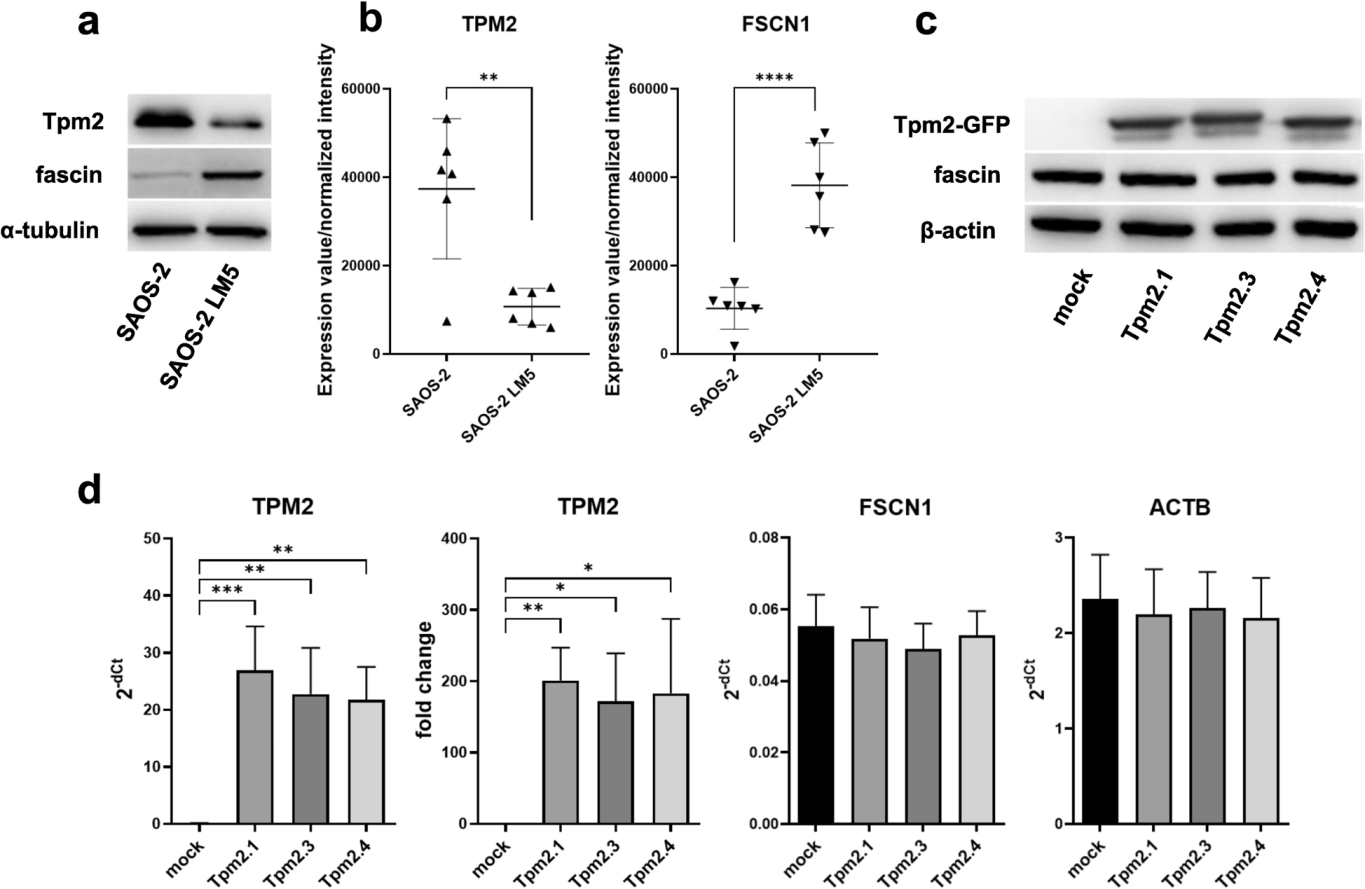
Tpm2 and fascin levels in non-metastatic SAOS-2 cells, highly metastatic SAOS-2 LM5 cells, and SAOS-2 LM5 cells overexpressing individual Tpm2 isoforms. **a** Tpm and fascin expression assessed by immunoblotting. **b** Normalized intensity values for *FSCN1* (probe A_33_P3411075) and *TPM2* (probe A_33_P3270599) retrieved from GEO dataset GSE66673 [62]. **c, d** Tpm2-GFP, fascin and β-actin levels in SAOS-2 LM5 cells after transient transfection with mock plasmid and plasmids coding for various Tpm2 isoforms assessed by immunoblotting (**c**) and qPCR (**d**). Significant differences (* p < 0.05, **p < 0.01, ***p < 0.001, ****p < 0.0001) are indicated. qPCR data represents mean ± SD from at least four independent experiments. Statistical analysis was performed using one-way ANOVA for multiple group comparisons and Student’s t-test for comparisons between two groups

Expression levels of fascin and Tpm2 were assessed by immunoblotting in parental SAOS-2 and metastatic SAOS-2 LM5 cells. As shown in the representative Western blot of cell lysates (Fig. 8a), fascin levels were elevated in metastatic SAOS-2 LM5 cells, whereas Tpm2 expression was reduced. This corresponds to the results of transcriptomic analyses of published datasets [62], which show increased *FSCN-1* and decreased *TPM2* mRNAs expression in SAOS-2 LM5 cells compared to parental SAOS-2 cells (Fig. 8b).

### Effects of Tpm2 isoforms on cell morphology and interaction of fascin with actin filaments in the SAOS-2 LM5 osteosarcoma cell line

**Fig. 9:**
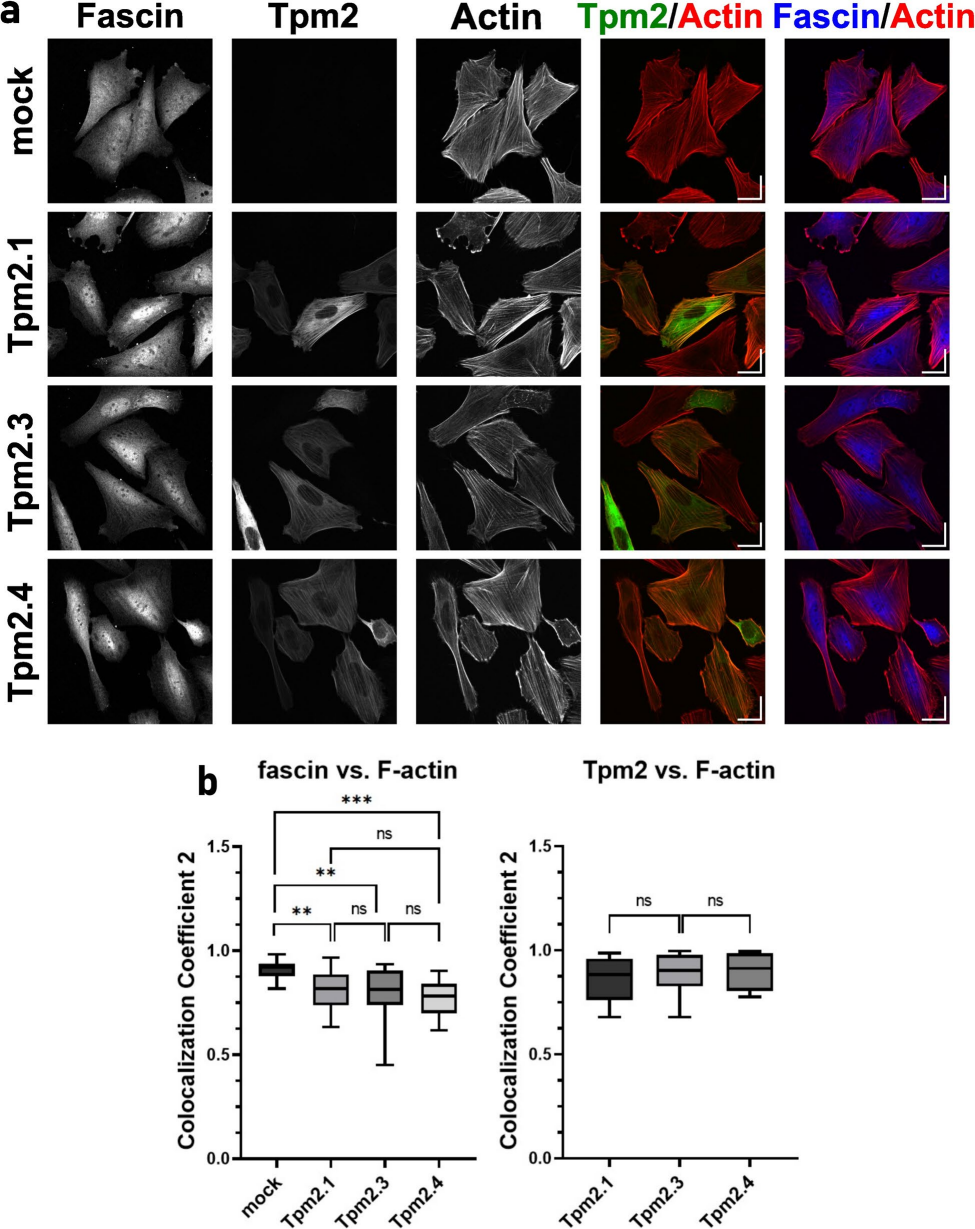
Effects of overexpression of cytoplasmic Tpm2 isoforms in metastatic SAOS-2 LM5 cell line. **a** Cellular localization of fascin, GFP-Tpm2 variants, and F-actin revealed by confocal fluorescence microscopy (scale-bar 10 µm). **b** Co-localization of fascin/F-actin, and Tpm2/F-actin determined by Co-localization Coefficient 2. For details see Materials and methods. Differences between the experimental groups were statistically significant (** p ≤ 0.01, *** p ≤ 0.001). Statistical analysis was performed using Tukey´s multiple comparison test

To confirm that Tpm2 isoforms modulate access of fascin to the actin cytoskeletal network at the cellular level, we transiently overexpressed GFP-fused Tpm2.1, Tpm2.3, and Tpm2.4 in metastatic SAOS-2 LM5 cells. We first confirmed the overexpression of all Tpm2 isoforms by immunoblotting and qPCR (Fig. 8c, d). All Tpm2 isoforms were overexpressed at similar level and no effect on fascin and actin expression was detected. Next, GFP-Tpm2 variants, fascin-1, and F-actin were visualized by fluorescence microscopy (Fig. 9a). Quantitative analysis of fascin/F-actin co-localization showed that fascin-free F-actin was less abundant in mock-transfected cells compared to cells overexpressing Tpm2.1, Tpm2.3, and Tpm2.4 (Fig. 9b), however specific effects of individual Tpm2 isoforms were not detected. Strong co-localization of all Tpm2 isoforms with F-actin was observed, confirming the actin-binding properties of Tpm2. In the cellular context, no differences in co-localization coefficient of Tpm2.1, Tpm2.3 and Tpm2.4 isoforms were observed (Fig. 9b), which most likely was due to high expression levels of all Tpm2 isoforms.

**Fig. 10:**
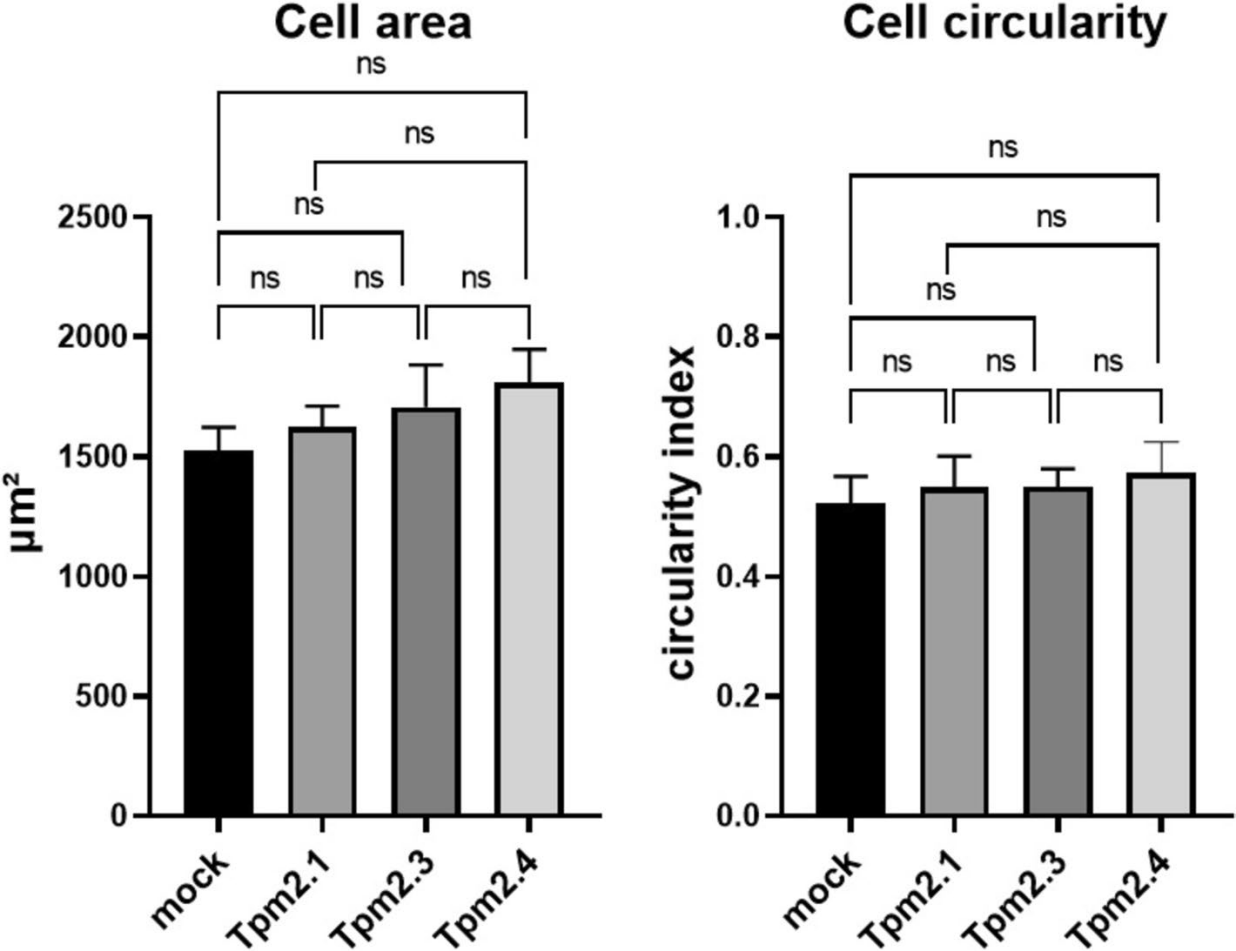
Overexpression of Tpm2 isoforms does not alter morphological parameters of SAOS-2 LM5 cells. Cell size and circularity assessed using microscopic images. At least 400 cells were evaluated in each group. Statistical analysis was performed using one-way ANOVA. No significant differences were observed between groups

**Fig. 11:**
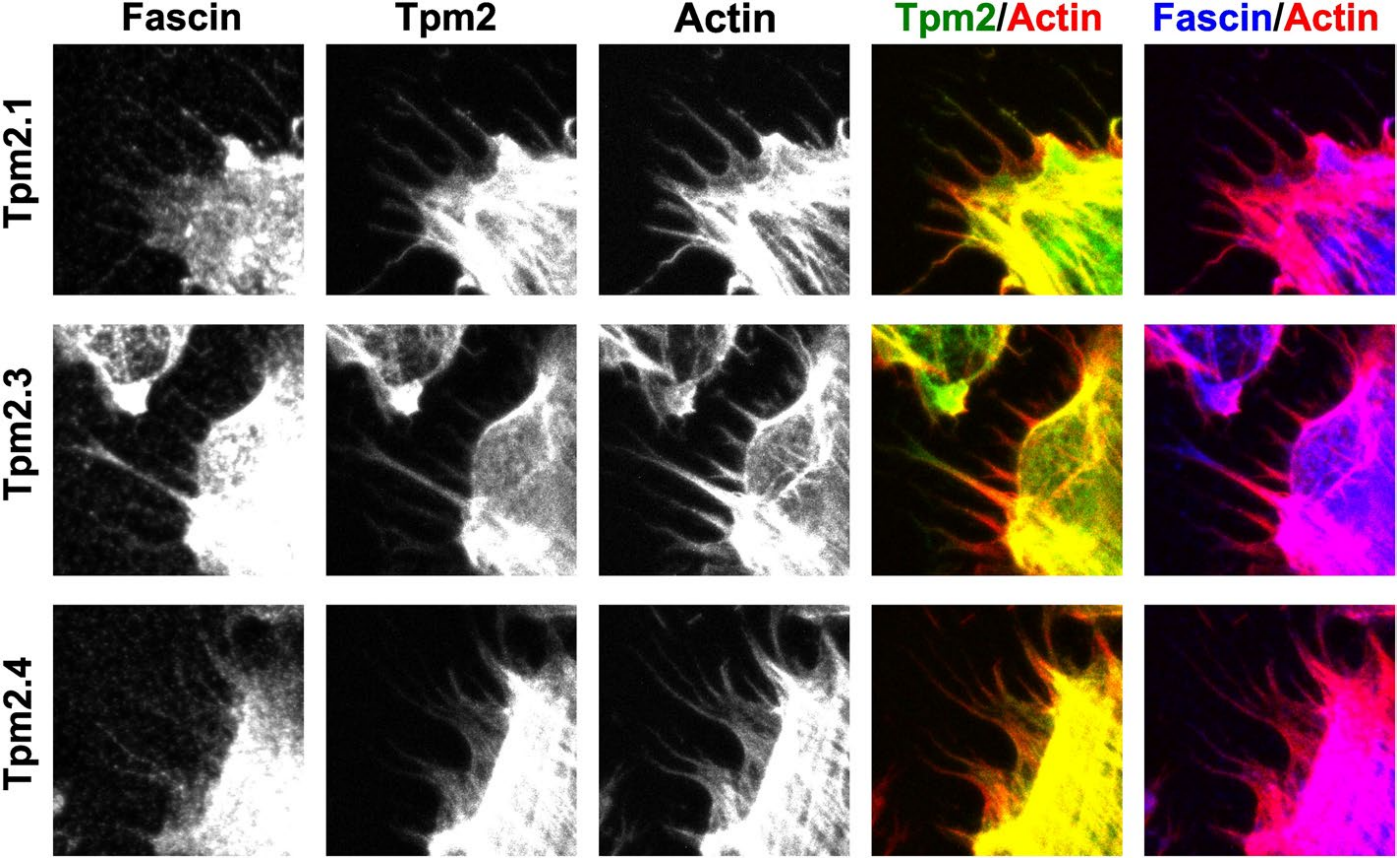
SAOS-2 LM5 cells overexpressing Tpm2 isoforms are capable of forming filopodia. Cellular localization of fascin, GFP-Tpm2 variants, and F-actin in filopodia revealed by confocal fluorescence microscopy

Since overexpression of Tpm2 can affect the actin cytoskeleton, a key determinant of cell morphology [63], we assessed the size and shape of SAOS-LM5 cells following transfection with individual Tpm2 isoforms. Only GFP-positive cells (mock and Tpm2.1-, Tpm2.3-, Tpm2.4-overexpressing cells) were included in the analysis. No significant differences in these parameters were observed among the experimental groups (Fig. 10). Interestingly, Tpm2-overexpressing cells were still capable of forming filopodia, where co-localization of Tpm2, fascin, and actin was observed (Fig. 11), indicating that their formation was not disrupted by elevated Tpm2 levels.

## Discussion

In various cancer types, distinct subsets of ABPs promote metastasis by regulating the organization and dynamics of the actin cytoskeleton. Since altered expression of fascin-1 and Tpm has been reported in cancer cells [2, 24, 27, 64], we used in vitro reconstituted actin filaments containing recombinant HMW Tpm2 isoforms (Fig. 1) and fascin-1 to investigate the mechanism by which Tpms regulate fascin.

Coating F-actin with each of the three bacterially produced, acetylated Tpm2 isoforms reduced fascin's actin affinity, though with distinct efficiencies. Tpm2.1 and Tpm2.3 caused a modest 1.3- to 1.6-fold decrease, whereas Tpm2.4 reduced fascin binding by nearly fourfold, correlating with its higher actin affinity. As Tpm2.4 binds to actin with the highest affinity, it appears to be the most effective at competing with fascin for actin. These results contrast with earlier reports suggesting that non-muscle HMW and LMW Tpms do not affect fascin’s actin affinity, despite differences in their own actin-binding properties [42]. Notably, those studies relied on isoform mixtures isolated from rat cultured cells, whereas our use of pure, recombinant Tpm2 isoforms reveals isoform-specific regulation of fascin. This highlights functional specialization among Tpm isoforms in modulating ABPs.

All Tpm2 isoforms strongly inhibited fascin-mediated actin bundling. To achieve ~ 40% bundling of Tpm-coated F-actin, approximately threefold higher fascin concentrations, compared to actin alone, were required. Interestingly, at saturating concentrations, fascin was able to overcome this inhibitory effect without displacing Tpm from the filament. A similarly strong inhibition of fascin-induced bundling was previously observed for skeletal muscle Tpm, but not for cytoplasmic isoforms [42]. These findings suggest that fascin activity is highly dependent on the specific Tpm isoform present. Since Tpms are sorted to different cell compartments [18, 21, 65], this isoform-specific regulation may represent a mechanism by which cells control fascin access to distinct actin structures.

Our observation that Tpm2 isoforms partially displace fascin from actin or prevent its binding supports a steric-blocking mechanism, previously proposed in the context of striated muscle Tpm-dependent regulation of fascin bundling activity [6]. However, the finding that both fascin and Tpms can be accommodated on the filament at high fascin concentrations suggests that a mixed mechanism may be at play. Such coexistence could involve direct interactions between fascin and Tpms, a possibility demonstrated in this work in vitro by a pull-down assay. Although these interactions remain to be verified under physiological conditions, the observed ability of fascin-1 to bind Tpm directly may help explain previous reports that overexpression of the non-muscle isoforms Tpm1.7 in B35 neuroblastoma cells promoted fascin recruitment to actin filaments in filopodia [44].

In the absence of high-resolution structures of F-actin–Tpm–fascin bundles, the molecular mechanism underlying Tpm-dependent regulation of fascin remains unclear. Our data show that fascin does not affect the actin-binding affinity of Tpm, indicating that Tpm2 can associate with actin filaments already decorated by fascin without restriction. Conversely, the presence of Tpm2 on the filament slightly reduces fascin’s affinity for F-actin and, at high Tpm2 concentrations, partially displaces fascin from the filament. Additionally, Tpm2 isoforms reduce the number of filaments incorporated into a bundle. These findings suggest that Tpm2 does not act merely as a competitor of fascin, but rather as an organizing factor that fine-tunes the protein composition and architecture of actin bundles.

The recent structure of fascin-decorated F-actin gives a clue on how F-actin accommodates both fascin and Tpm. The fascin-binding site on the actin filament comprises two actin subunits that are adjacent along the filament axis, engaging subdomain 1 and the D-loop, regions that are exposed on the outer edge of the filament [16]. By binding to actin, Tpm chains extend along the filament’s inner domain [66], providing sufficient space for fascin to bind. Certain Tpm isoforms were shown to adopt different orientations on the filament [67], which might be the major reason of differences between the isoforms in regulation of fascin interactions with actin. To cross-link two parallel actin filaments, fascin engages two actin-binding sites, which requires conformational flexibility of fascin adopting the helical structure of the filament [16]. It is possible that by coating the filament, Tpm stabilizes the helical twist of the filament and by restricting the flexibility it controls the number of fascin molecules present in the bundle. It appears that the presence of Tpm increases the cooperativity of fascin binding to actin filaments, despite reducing its overall affinity. This suggests that fascin can overcome the conformational stabilization imposed by Tpm, possibly by inducing a specific actin conformation that is propagated along the filament. Verification of this possibility needs further structural studies in which Tpm will be included as a central regulator of the filament.

. To investigate the effect of Tpm2 overexpression on the fascin–actin interaction in a cellular context, we selected SAOS-2 LM5 cells. These highly metastatic osteosarcoma cells were derived from parental, low metastatic SAOS-2 cells by repeated in vivo selection of metastasis efficient cells [55]. It has been previously shown that parental SAOS-2 cells express both fascin and Tpm2 at the protein level [36, 68, 69]. Our analysis revealed increased expression of fascin and decreased expression of Tpm2 in SAOS-2 LM5 cells compared to parental SAOS-2 cells, consistent with the proposed roles of fascin as a metastasis promoter [32–35] and Tpm2 as metastasis suppressor [70–72].

Overexpression of the *TPM2* protein products, Tpm2.1, Tpm2.3, or Tpm2.4, in the metastatic SAOS-2/LM5 cells resulted in reduced co-localization of fascin with actin, suggesting a partial displacement of fascin from actin fibers. Given the roles of Tpm2 and fascin in actin cytoskeleton dynamics, downregulation of *TPM2* coupled with upregulation of *FSCN1* may collectively contribute to cytoskeletal remodeling, enhancing cancer cell motility, invasiveness, and metastatic potential. Forced expression of fascin and knock-down of Tpm2 has been previously linked with enhanced migration/invasion in various cancer cell lines [36, 47, 73–76].

It should be noted, however, that the cellular model used in our study has certain limitations. Our in vitro experiments demonstrated that the interaction between fascin and actin, as well as fascin-mediated actin bundling activity, depends on the concentrations of both fascin and Tpm2. Tpm2 displaces fascin from actin filaments only at high Tpm:fascin ratios. At the cellular level, the situation is more complex, as other ABPs may influence the interactions among Tpm2, fascin, and actin. Transient transfection was employed to achieve high levels of Tpm2 isoforms in SAOS-LM5 cells, with the aim of demonstrating that Tpm2 can also displace fascin from actin fibers in a cellular context. At the RNA level, the approximate *TPM2:FSCN1* transcript ratio of 4:1 in parental SAOS-2 cells is reversed to 1:4 in the metastatic SAOS-2 LM5 variant (Fig. 8b). Following transfection, we achieved over 100-fold overexpression of *TPM2* mRNA in SAOS-2 LM5 cells, resulting in a *TPM2:FSCN1* ratio significantly higher than that observed in parental SAOS-2 cells. Such high (and potentially non-physiological) levels of Tpm2 may lead to dosage-related artifacts, including altered localization, interactions, folding, or protein aggregation [77], and therefore, the results must be interpreted with caution. Nevertheless, we observed strong co-localization of exogenous Tpm2 with actin, suggesting that its function as an ABP was preserved. High level of Tpm2 can also displace other ABPs in actin filaments (including endogenous tropomyosins) that may affect fascin-actin interactions and formation of actin structures in cells.

Fascin-dependent bundling of F-actin is important in the formation of plasma membrane protrusions such as filopodia [8, 78]. Interestingly, formation of filopodia was not disrupted in Tpm2-overexpressing cells. Tpm2, fascin, and actin co-localize within these fine cellular structures suggesting that Tpm2 and fascin can coexist on actin filaments as observed in our in vitro assay. However, due to the limited resolution of our imaging approach, we cannot exclude the possibility that these proteins are localized on distinct actin filaments within filopodia. Further high-resolution techniques would be required to clarify their precise spatial arrangement.

Another consequence of altered Tpm expression is the impaired ability of the actin cytoskeleton to sense the mechanical properties of the external environment, a process known as rigidity sensing. Downregulation of HMW tropomyosins, including Tpm2.1, is an early event in cellular transformation (e.g., following activation of oncogenic Ras or Src), leading to the disruption of stress fibers and enabling oncogenic behaviors such as anchorage-independent growth [45]. Tpm2.1 is a critical component of rigidity-sensing complexes, sarcomere-like contractile units that generate traction forces on the extracellular matrix. In transformed cells, reduced Tpm2.1 expression directly results in the loss of matrix rigidity sensing, allowing these cells to bypass normal rigidity-dependent growth constraints and proliferate on soft substrates [79–81]. Whether fascin-1 is present in these contractile units, or whether Tpm2.3 and Tpm2.4 play any modulatory role in this process, remains to be determined.

The involvement of Tpm2 isoforms and fascin-1 in cancer development and metastasis makes them promising candidates for prognostic markers and potential therapeutic targets. In recent years, fascin-1 inhibitors have shown potential in slowing cancer progression in vitro and in preclinical models [41, 82–85]. Since Tpm isoforms are abundantly, yet specifically expressed in all cells and co-polymerize with the majority of actin filaments [25], understanding the mechanisms underlying Tpm-dependent regulation of fascin activity is essential for the development of effective anti-cancer therapies.

## Data Availability

The datasets supporting the conclusions of this article are available in the RepOD repository, https://repod.icm.edu.pl/dataset.xhtml?persistentId=doi%3A10.18150/8IL2AR

## Abbreviations

*TPM2*: Tropomyosin 2 gene
*TPM2.1, TPM2.2. TPM2.3, TPM2.4*: cDNA encoding isoforms of tropomyosin 2
Tpm2: General protein product of *TPM2* gene
Tpm2.1, Tpm2.2, Tpm2.3, Tpm2.4: Protein isoforms of *TPM2*
*FSCN*1: Fascin-1 gene
OSA: Osteosarcoma
ABP: Actin binding protein
HSC: High-speed centrifugation
LSC: Low-speed centrifugation
TRC: Tetramethylrhodamine cadaverine
